# Partial melting of deeply subducted eclogite from the Sulu orogen in China

**DOI:** 10.1038/ncomms6604

**Published:** 2014-12-17

**Authors:** Lu Wang, Timothy M. Kusky, Ali Polat, Songjie Wang, Xingfu Jiang, Keqing Zong, Junpeng Wang, Hao Deng, Jianmin Fu

**Affiliations:** 1 State Key Laboratory of Geological Processes and Mineral Resources, China University of Geosciences Wuhan, Wuhan 430074, China; 2 Center for Global Tectonics, China University of Geosciences Wuhan, Wuhan 430074, China; 3 Three Gorges Research Center for Geohazards, Ministry of Education, China University of Geosciences Wuhan, Wuhan 430074, China; 4 Department of Earth and Environmental Sciences, University of Windsor, Windsor, Ontario, Canada N9B 3P4; 5 School of Earth Sciences, China University of Geosciences Wuhan, Wuhan 430074, China

## Abstract

We report partial melting of an ultrahigh pressure eclogite in the Mesozoic Sulu orogen, China. Eclogitic migmatite shows successive stages of initial intragranular and grain boundary melt droplets, which grow into a three-dimensional interconnected intergranular network, then segregate and accumulate in pressure shadow areas and then merge to form melt channels and dikes that transport magma to higher in the lithosphere. Here we show, using zircon U–Pb dating and petrological analyses, that partial melting occurred at 228–219 Myr ago, shortly after peak metamorphism at 230 Myr ago. The melts and residues are complimentarily enriched and depleted in light rare earth element (LREE) compared with the original rock. Partial melting of deeply subducted eclogite is an important process in determining the rheological structure and mechanical behaviour of subducted lithosphere and its rapid exhumation, controlling the flow of deep lithospheric material, and for generation of melts from the upper mantle, potentially contributing to arc magmatism and growth of continental crust.

## Abstract (web summary)

Natural examples of eclogite-derived migmatite were previously unknown. Here, the authors show evidence of high-pressure melting of eclogite in the Sulu orogen of China, and suggest the melts represent an exhumed example of flow channels in the lower crust, possibly a source of syn-exhumation magmas.

## Graphical abstract

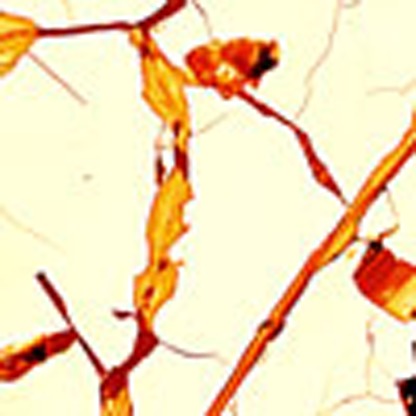

Documenting partial melting processes in the deep crust and mantle is important for understanding deep lithospheric rheology, as well as the origin and genesis of arc magmas, continental and oceanic crust^1^. Numerous studies have described the partial melting processes in felsic gneiss-forming migmatites^1^,^2^,^3^,^4^, some of which may generate melts that coalesce to form plutons^5^,^6^,^7^. However, the partial melting of eclogite at the microscopic to field scales has never been clearly documented. If the partial melting of eclogite can be clearly demonstrated, then there will be clear evidence that subducted oceanic slabs and deeply subducted continental crust can melt. It would then be important to determine how these melts escaped from their nucleation points to form networks, dikes or plutons that could buoyantly rise to higher in the crust, contributing to arc magmatism and the formation of continental crust.

The Sulu orogen of eastern China was born during rifting of the Rodinian supercontinent about 800 Myr ago, leading to formation of an ocean basin, and grew during subduction of this oceanic lithosphere, evolving to maturity with the collision of the Yangtze and North China cratons and deep subduction of intervening continental material^8^. Most of the orogen preserves greenschist- to amphibolite-facies mineral assemblages, but detailed studies^9^,^10^ documenting the U–Pb ages of zircons with coesite inclusions from granitic gneiss and marble cropping out together with coesite-bearing eclogite indicate earlier ultrahigh pressure (UHP) metamorphism in the Late Triassic at about 230 Myr ago^11^,^12^,^13^. Intergranular coesite and clinopyroxene+rutile+apatite exsolutions within garnet in eclogite at Yangkou in the central Sulu orogen^14^,^15^ indicate subduction of this eclogite to ~200 km. Most researchers attribute the origin of the eclogites to the subcontinental lithospheric mantle (SCLM)^16^. Felsic gneiss, the country rock of UHP eclogites in the whole Sulu orogen, has experienced decompressional partial melting during exhumation^7^,^17^,^18^,^19^,^20^. Some evidence for limited partial melting of UHP eclogite and quartzite has been proposed based on the presence of multiphase solid (MS) inclusions within UHP minerals and anatectic zircons and interstitial cuspate K-feldspar along quartz grain boundaries^21^,^22^. In addition, high-pressure experimental petrology has demonstrated that phengite-bearing eclogite can undergo partial melting during decompression under conditions of *P*=1.5–2.0 GPa and T≤800–850 °C (refs 23, 24). However, before this study, no field evidence has been documented to show multiscale partial melting of eclogite, resulting in eclogitic migmatites that feed larger-scale felsic dikes, which intrude the continental crust.

This contribution represents the first documented example of field, microstructural, geochemical and geochronological evidence in the world for partial melting of deeply subducted eclogite, using exposures in the UHP Sulu Orogen, China, showing that these mafic rocks were subducted to >120 km depth at 230 Myr ago, then partially melted during their early retrograde path to the surface 228–219 Myr ago. Several different stages of melt segregation are delineated, starting from the formation of initial melt droplets along grain boundaries to the coalescence of these melt droplets along an interconnected three-dimensional (3D) intergranular network of microveins, then along foliation planes and extensional shear zone surfaces. The melt then flowed into the thin macroscopic veins that ballooned in low-stress areas such as pressure shadows, particularly along fold hinges between the units with different competence. Melt-enhanced deformation next aided the melt pockets to merge into 1–2-m wide melt channels spaced every 10–20 m in the host eclogite, where melts form about 50% of the rock, and eclogitic residue forms boudins and isolated complex folds. The melt channels eventually combine forming several-metre-wide dikes, which served as conduits bringing the melt to higher levels of the lithosphere. The partial melting of deeply subducted eclogite has several very important implications. First, the melt channels that are spaced every 10–20 m represent significant seismic velocity anomalies that should be accounted for in analysis of seismic data for geophysical models of the lithosphere^25^,^26^ Second, the process of melting and melt aggregation from the subgrain scale to the pluton-feeding dike scale can be shown to be closely linked with structural processes, and the melt-enhanced deformation shows how deformation, melting and metamorphism are closely linked^27^,^28^,^29^. Third, recognizing melt channels in eclogite is important for understanding mid-to-lower crustal and mantle flow: the melt channels have a much lower viscosity than the surrounding eclogite and can accommodate large amounts of strain, allowing translation of intervening blocks. Melt-enhanced deformation and flow in melt channels documented here may be an exhumed example of the deep crustal flow channels accommodating continental escape from continental collision zones such as eastern Asia^30^,^31^,^32^, and also, be important in lubricating the borders of slices of UHP blocks, allowing the UHP rocks to be rapidly exhumed along the low-viscosity flow channels^33^,^34^.

## Results

### The General's Hill eclogite

**Figure 1:**
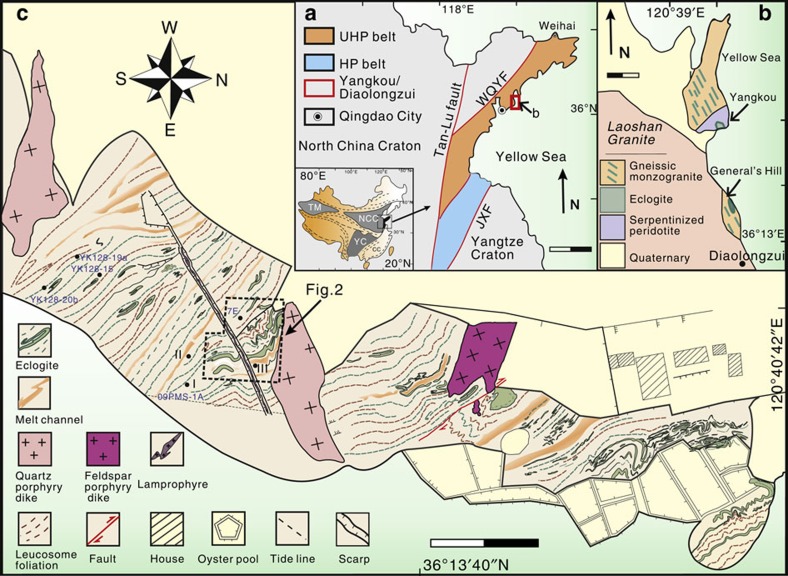
Geological map of Yangkou bay and General's Hill. (**a**) Simplified geological map of the Sulu orogen and its location in China. Scale bar, 100 km at 50-km intervals. (**b**) Geological map of Mt. Laoshan and the structural setting of Yangkou Bay and General's Hill^35^. Scale bar, 1 km at 0.5 km intervals. (**c**) Map of continuously exposed coastal outcrops at General's Hill. Scale bar, 30 m at 15–m intervals. Our detailed 1:1,500 scale mapping delineates strongly foliated and complexly folded retrogressed eclogite, cut by channels of dominantly felsic leucosome. The most weakly retrogressed part of the eclogite body consists of strongly foliated isoclinally folded eclogitic gneiss, interlayered with foliated felsic leucosome and retrogressed eclogite (now garnet-bearing amphibolite). In other places the eclogite is preserved as sheared boudins with leucosome and quartz veins in pressure shadows of the eclogitic boudins. Mapping by L. Wang, T. Kusky, S. J. Wang, J. P. Wang and Y. Ding.

**Figure 2:**
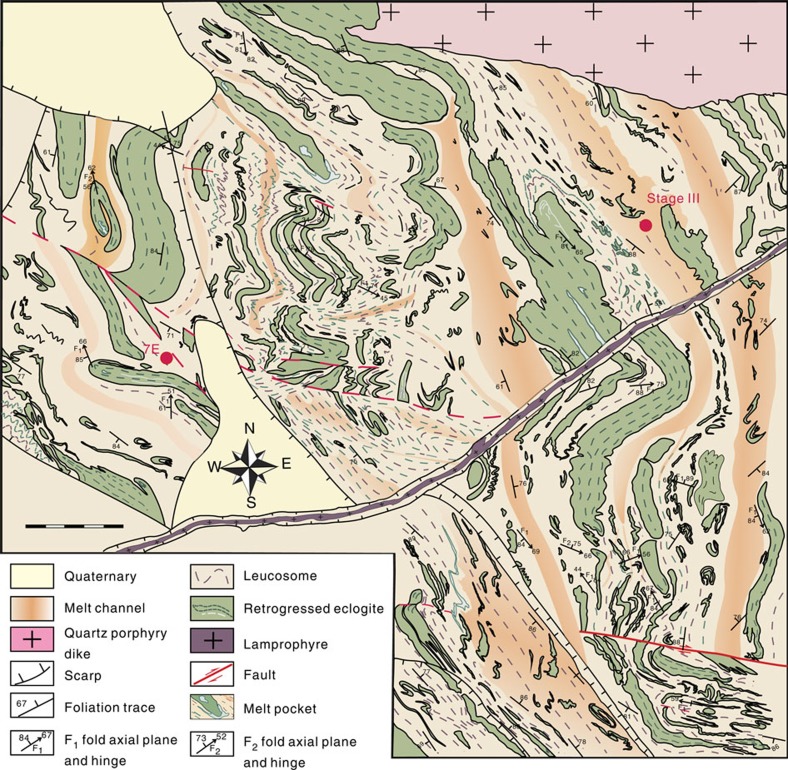
Structural map of melt channels at General's Hill. Multiple leucosome veins, melt pockets and melt channels merging to form dikes at General's Hill are shown within the map (see Fig. 1c for location, noting the difference in orientation of maps). Most eclogite is retrogressed into garnet-bearing amphibolite deformed into rootless isoclinal and less-common sheath folds, and disaggregated into boudins surrounded by leucosome. Their strong foliation is mostly defined by biotite and amphibole. These folds typically have thicker hinges than limbs (ptygmatic folds) or are strongly sheared and boudinaged along their limbs. In some locations, the hinges of the isoclinal folds are also sheared, thinned and broken into boudins with felsic leucosome flowing into the boudin necks and pressure shadows behind fold hinges of layers with stronger competence than surrounding layers. Once the melt was present in these regions, the melt enhanced the deformation, further localizing strain and melt concentration in these locations. The melts appear as a leucocratic matrix and flows around the retrogressed eclogite layers. The axial planes are almost coincident with the NW-striking foliation that dips steeply to the NE. 7E, geochronological sample location of melted eclogite as shown in Fig. 7c (stage II). Stage III, location of last stage of melting process as shown in this figure. Mapping by T. Kusky, L. Wang, S. J. Wang, J. P. Wang and Y. Ding. Original scale 1:2,000; scale bar, 5 m at 1-m intervals. Base map provided by Laoshan National Park.

The General's Hill eclogite is located in the central Sulu orogen on the Shandong Peninsula, eastern China, a few kilometres south of the famous exposures of the world’s most deeply subducted and exhumed eclogites at Yangkou (Fig. 1a–c) and about 1 km north of a village called Diaolongzui. After initial field observations suggested that the eclogites at Yangkou and General's Hill may have experienced partial melting, we documented the processes at several different scales. The first step was targeted 1:1,500–1:200 scale mapping (Figs 1c and 2) of continuously exposed coastal outcrops of deeply subducted and exhumed rocks at Yangkou Bay^35^,^36^, and newly discovered outcrops near General's Hill, Diaolongzui village (Fig. 1b,c). The exposures consist of strongly foliated and complexly folded retrogressed eclogitic migmatite (Fig. 2) with the partial melt veins feeding channels of dominantly felsic leucosome (leucosome is the light-colored part of migmatite, consisting mostly of K-feldspar+plagioclase+quartz, interpreted to be the crystallized melts derived from the partially melted eclogite, which is now the residue, the darker part of the migmatite). Next, we used a multiscale multidisciplinary approach including structural analysis, microstructural petrology and scanning electron microscope (SEM) fabric analysis, geochronology and geochemistry, which verify that the Yangkou and General's Hill eclogites underwent partial melting, and the melts migrated out of the system to interact with other rocks and melts in the deep lithosphere.

Outcrop and microscale structures that show the progressive process of partial melting of eclogite are systematically documented. Three typical stages characterize the different processes of partial melting, demonstrating how melt starts from miniscale droplets along grain boundaries, coalesce into veins that merge into melt channels, which then feed dikes where eclogite-derived melts interact with melts derived from the country gneisses, and transport large volumes of magma from deep levels of the crust/mantle, to higher in the crust (Fig. 1c).

#### Stage I

**Figure 3:**
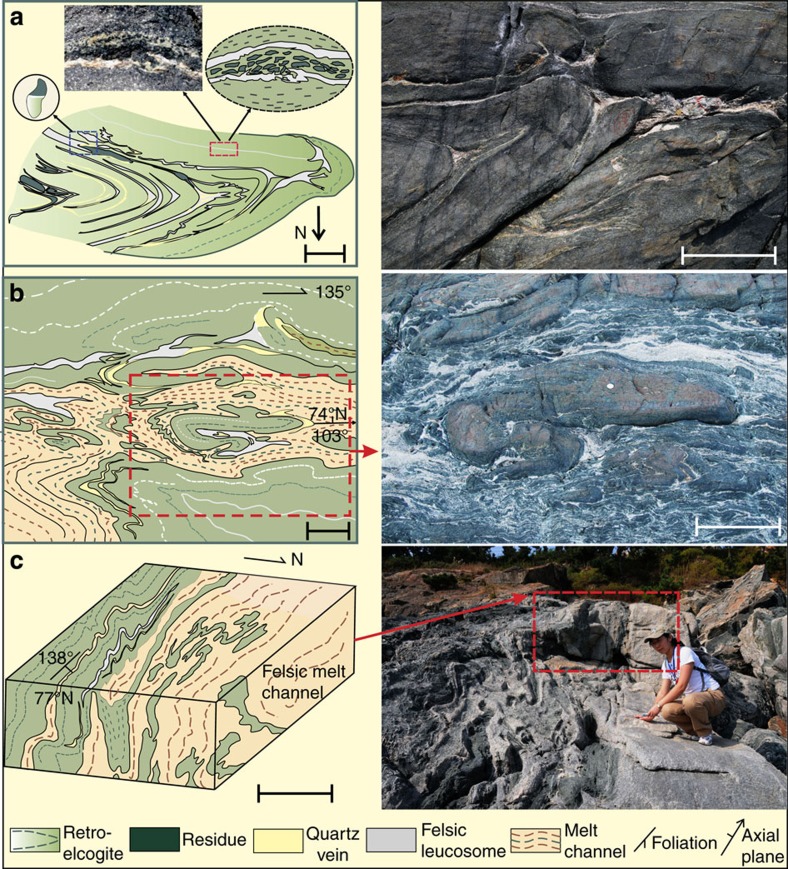
Sketches and associated photographs showing the partial melting processes. (**a**) Stage-I. Early stage of partial melting, finger-shaped leucosome starts to aggregate within the hinge of the isoclinal eclogite fold. Scale bar, 5 cm. Sketches or field photo pointed to the blue and red box represent enlarged area where the boxes are. Field photo connected to the red box by the black arrow shows the irregular boundary between leucosome and residue that supports a magmatic partial melting genesis, scale bar in this photo is 1 cm across. (**b**) Stage-II. Medium stage of partial melting, melt channels (leucosome) interlayered and flowing surrounding the sheared folded eclogite. Scale bar, 10 cm. Red dashed box represents the same range of the field photo on its right column. (**c**) Stage-III. Mature stage of partial melting, melt aggregates into larger veins and then forms felsic dikes. Scale bar, 100 cm. The red box represents the same spatial range as the field sketch.

**Figure 4:**
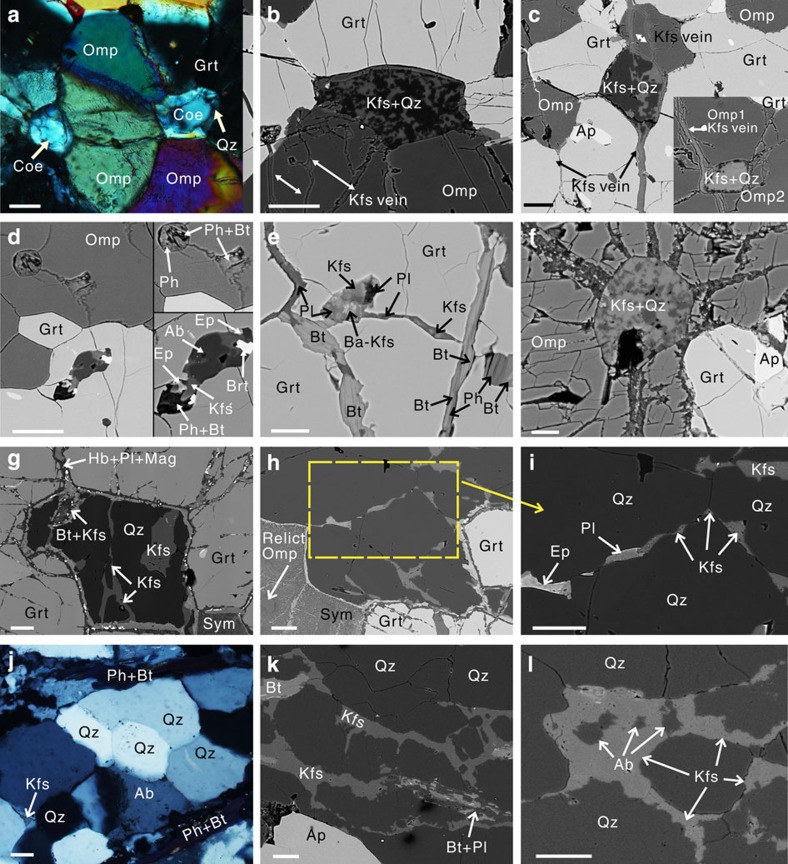
Microphotographs of partially melted eclogites and leucosome (melt) in the Sulu orogen. (**a**–**d**) YK05-2a, UHP stage-1 eclogite (Grt+Omp+Coe/Qz, Ph free) from Yangkou. (**a**) Intergranular coesite with higher relief and surrounded by retrogressed quartz with lower relief. (**b**,**c**) Rounded interstitial pod composed of Kfs+Qz with fractures cross-cutting and filled with Kfs. (**d**) MS inclusion composed of Ph+Bt and Kfs+Ab+Brt+Ep in garnet with veinlet and inclusion of Ph+Bt connected, indicating *in situ* partial melted phengite inclusions within garnet and omphacite grains. (**e**) YK12-3a, phengite-bearing Qz-eclogite (Grt+Omp+Qz+Ph) from Yangkou, phengite *in situ* dehydration melting, forming a MS inclusion of Kfs+Pl+Ba-Kfs within garnet, connecting with veinlets filled by Bt+Kfs+Pl next to the phengite. (**f**) YK12-3a, MS inclusion of Kfs+Qz within omphacite with radial fractures. (**g**) YK128-15, eclogitic residue with mineral assemblage of Grt+Omp+Qz from General Hill; sym represents symplectite replacing previous omphacite. MS inclusion of Kfs+Qz developed within garnet, surrounded by Hb+Pl+Mgt rim. (**h**,**i**) YK128-15, cuspate veinlets of Kfs+Pl+Ep (melt droplets) with low dihedral angles, and form ‘strings of beads’ along grain boundaries between quartz and at triple junctions. (**j**) 09PMS-1A, leucosome sample from Fig. 1d, inset photo of stage I, clear triple junction texture developed at grain boundaries of quartz grains, with K-feldspar filling in the triple junction. (**k**,**l**) YK128-16a, leucosome sample, more advanced stages of partial melting where the melt droplets (Kfs+Pl) along grain boundaries have merged and formed an interconnected 3D network along grain boundaries and micro-cracks enabling the melt to drain out of the intergranular areas into low-stress regions in the leucosome. Scale bars, 15 μm in **e**,**f** and 50 μm in **a**–**d** and **g**–**l**. **a** and **j** are microscope photographs under cross-polarization, the rest are all back-scattered electron images. Ab, albite; Ap, apatite; Bt, Biotite; Coe, coesite; Grt, garnet; Hb, hornblende; Kfs, K-feldspar; Mag, magnetite; Omp, omphacite; Ph, phengite; Pl, plagioclase; Qz, quartz.

The earliest stages of partial melting are preserved as beaded droplets of leucosome composed of quartz-plagioclase-K-feldspar along grain boundaries in the eclogite, as revealed by high-power photomicroscope and SEM imagery (Figs 3a and 4h,i). Near fold hinges and along cross-cutting extensional shear zones these droplets of leucosome merge to form veins 2–3 mm wide along the foliation of the folded eclogite. These thin veins then merge along these structures where the melt flowed towards low-pressure regions in hinges of isoclinal folds (Fig. 1c, location I; Fig. 3a, stage I). The leucosome is composed of quartz and minor plagioclase+K-feldspar+Phengite/Biotite. The quartz grains have wavy extinction under polarized light and contain abundant fluid inclusions. The quartz grains are anhedral with clear triple junction texture, indicating high temperature recrystallization without high shear stress (Fig. 4j). The irregular boundary between leucosome and residue supports a magmatic partial melting genesis^37^ (Fig. 3a). The residue has various mineral compositions that show different stages of retrogression at different sample locations since the process of partial melting is inhomogeneous and is accompanied by different levels of retrogression. Phengite and mica disappear in the residue. The least retrogressed eclogite assemblage consists of Grt+Omp+Qz+Rt+Sym (Hb+Pl), with symplectite forming a vein-net texture cutting through garnet grains, with Hb+Pl+Mgt within the veins (Fig. 4g). All the mineral abbreviations in this paper follow the standard of Whitney & Evans^38^. Garnet and Omphacite are typically strongly elongated. The garnet amphibolites have garnet partially preserved due to retrogression, and omphacite is nearly completely replaced by symplectite composed of fine grained plagioclase+hornblende.

#### Stage II

Melt begins to aggregate within thin veins and extensional shear bands, then it starts to flow along foliation planes in the eclogite and becomes interlayered with the residue layers and symplectite of the retrogressed minerals from the eclogite. The leucosome forms melt channels where it occupies about 50% of the rock, bounded by relatively less retrogressed eclogite. Most channels are about 0.5–1 m wide and spaced every 10–20 m. The leucosome flows and becomes folded together with the remnant rootless isoclinal folds of eclogite and is further concentrated within fold hinges, indicating that the partial melting and deformation were contemporaneous, with the melt enhancing the deformation, and the deformation aiding the concentration of melt in low-stress regions (Fig. 1c, location II; Fig. 3b, stage II).

In stage II, the mineral composition of the leucosome is quartz+biotite/phengite+plagioclase+K-feldspar+minor amphibole, epidote and apatite. There is no clear shape-preferred orientation, and garnet has gradually reduced from the residue, replaced by needle-shaped or clustered amphibole, plagioclase and biotite. The eclogite shows more intensive symplectite developed along the garnet grain boundaries. The grain size of garnet decreases and the symplectite forms a cross-cutting network containing small round-shaped garnet relicts.

#### Stage III

In this stage, the leucosome becomes more concentrated and finally forms thick felsic dikes, which may have interacted with melts derived from the country gneisses (Fig. 1c, location III; Fig. 3c, stage III). The eclogite is totally retrogressed and epidote amphibolite facies residue dominates the outcrop, becoming another end-member of this partial melting process. In this stage, the original eclogite is completely reconstituted into two new rock types: felsic dikes and cpx-bearing amphibolite.

Mineral assemblages of felsic leucosome are coarser grained, consisting of biotite/phengite+quartz+plagioclase+K-feldspar, showing no clear shape-preferred orientation, but instead a euhedral to sub-euhedral granular texture. The quartz grain boundaries are different from stage I and II, they are very rich in fluid inclusions, characterized by dynamic recrystallization and subgrain boundary migration, indicating a high temperature fabric. The epidote-bearing amphibolite is composed of amphibole+plagioclase+minor epidote and minor ilmenite, apatite and sphene.

**Figure 5:**
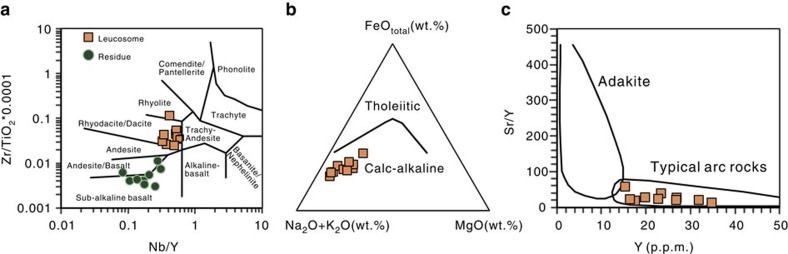
Geochemical plots of ten rock pairs of leucosome and residue. These samples were collected in the region between two quartz porphyry dikes of Fig.1c. (**a**) Geochemical plot of Yangkou Bay leucosome and residue samples in the Nb/Y versus Zr/TiO_2_ ratio diagram from^58^. (**b**) Distribution of Yangkou Bay leucosome samples in the FeO_total_-Na_2_O+K_2_O-MgO ternary diagram from ref. 59, wt.%, weight percent. (**c**) Diagram for Sr/Y ratios versus Y contents (p.p.m.) for the Yangkou Bay leucosomes. The fields for adakites and typical arc rocks are after Defant and Drummond^51^. See original data from Supplementary Data Set 1 and 2.

Whole-rock and rare earth element (REE) analyses of the leucosome indicate that they are dacitic to rhyolitic in composition with calc-alkaline arc affinities (Fig. 5, see Supplementary Data Set 1 and 2).

### Microanalysis and P–T–t path

**Figure 6:**
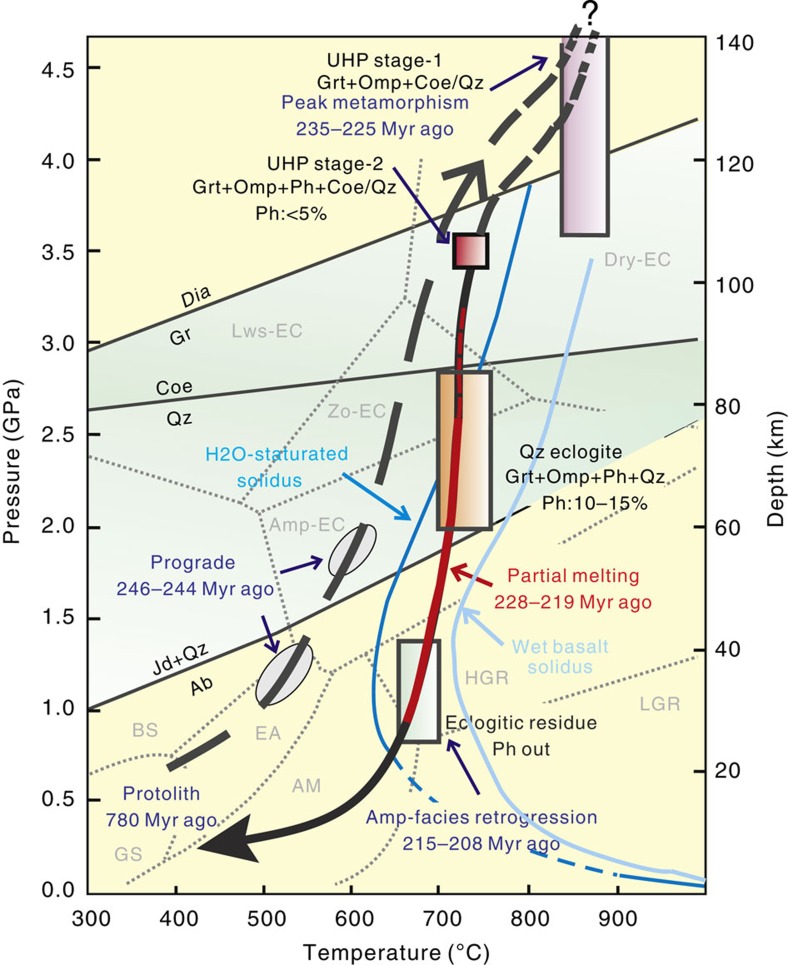
P–T–t path of the UHP eclogite and eclogitic residue in the Yangkou and General's Hill, Sulu Belt. Ages of prograde, peak metamorphism and amphibolite-facies retrogression stages are from ref. 15. The ages of the protolith and partial melting are from this study (original data shown in Supplementary Data Set 4). Eclogite from UHP stage-1 contains no phengite; however, phengite in eclogite from UHP stage-2 to quartz eclogite facies rises to 5–15%. These three types of eclogite samples were taken from Yangkou Bay, eclogitic residue sample is taken from General's Hill. Our P–T estimation is based on the geobarometer of Waters & Martin^41^ and the geothermometers of Ravna^40^ and Green & Hellman^42^. Original EMP (electron microprobe) data are shown in the Supplementary Data Set 3. The P–T–t path is modified from Zhang and Liou^60^; Liu and Liou^13^. Petrogenetic grids, subdivision of eclogite-facies and reaction curves diamond=graphite, coesite=quartz and jadeite+quartz=albite are from Bundy^61^, Oh and Liou^62^, Holland^63^ and Bohlen and Boettcher^64^, respectively. Dark blue curve of the water-saturated basalt solidus is from Lambert & Wyllie^65^ and light blue curve of the wet solidus is from Schmidt & Poli^66^. AM, amphibolites facies; AM, amphibolites facies; Amp-EC, amphibole eclogite facies; BS, blueschist facies; Dry-EC, dry eclogite facies; EA, epidote amphibolites faciesl; GS, greenschist facies; HGR, high-pressure granulite facies; LGR, low-pressure granulite facies; Lws-EC, lawsonite eclogite facies; Zo-EC zoisite eclogite facies.

Detailed petrological phase analyses were made on UHP eclogites and residue from Yangkou Bay and General's Hill. At least four stages of metamorphism are identified and their metamorphic pressure–temperature (P–T) conditions are estimated based on electron probe data (Supplementary Data Set 3) and shown as a P–T–time (P–T–t) path in Fig. 6. Microstructural evidence of partial melting of eclogite is also evident in these four types of eclogite (Fig. 4).

UHP stage-1 eclogite: mineral assemblage is Grt+Omp+Coe/Qz+Rt, coesite occurs as intergranular crystals (Fig. 4a) and as inclusions in garnet and omphacite. Coesite with higher relief has partially been transformed to quartz with lower relief in the rim, which shows a typical palisade texture. Coesite was identified microscopically and confirmed by Raman spectroscopy (Supplementary Fig. 1). The mineral assemblage is similar to the type of massive eclogite reported elsewhere in the Dabie–Sulu UHP metamorphic belt^39^. The UHP stage-1 metamorphic temperature is estimated to have been 834–890 °C based on the geothermometer of Ravna^40^, when the pressure is set at 3.5–4.5 GPa (Fig. 6). The lower limit for the pressure is 3.5 GPa, since intergranular coesite occurs in both two types of UHP eclogite (UHP stage-1 and 2 as described in the next paragraph). The maximum pressure is set as 4.5 GPa for this study in Fig. 5, although the possible maximum pressure can reach to 6 GPa (*T*=970 °C, see Supplementary Data Set 3) based on reports of mafic slabs that were subducted to >200 km depth from Yangkou Bay^15^. MS inclusions consisting of Kfs+Ab+Ep+Brt+Ph+Bt are present in garnet (Fig. 4d). The MS inclusion illustrated is connected by a veinlet with another phengite inclusion in omphacite, which is breaking down into biotite and fills in melting pods along the veinlet. This is clear evidence for *in situ* partial melting of phengite inclusions in UHP eclogite. Intergranular multi-solid phase of Kfs+Qz is preserved in this stage with the quartz replacing some of the previous intergranular coesite and the Kfs filling fractures, which cut through the eclogite, or fill in the radial cracks surrounding the intergranular Kfs+Qz (Fig. 4b,c). This indicates that the Kfs veins are a later stage of the melting process, carrying fluid that promotes the retrogression of coesite to quartz.

#### UHP stage 2 eclogite

In this stage, the mineral assemblage is Grt+Omp+Qz/Coe+Ph (<5%)+Rt. With the appearance of phengite, the amount of preserved intergranular coesite is obviously reduced. Phengite is developed along the penetrative foliation planes in the eclogite. The mineral assemblage is similar to the type-2 foliated eclogite reported in the Dabie–Sulu UHP metamorphic belt^39^. The P–T estimation is *P* (average)=3.5 GPa and *T* (average)=733 °C based on the barometer of Waters and Martin^41^ and the thermometer of Green and Hellman^42^. Phengite has 3.5–3.6 Si atoms per formula unit. MS inclusions similar to those in UHP stage-1 eclogite are present in stage-2 eclogite as well.

#### Quartz eclogite

The mineral assemblage in this stage is Grt+Omp+Qz+Ph (~10–15%)+Rt, with higher amounts of phengite. Coesite has completely changed to quartz in this stage. Our P–T estimation for this stage is *P*=1.5–2.8 GPa (lower and upper pressure limits for eclogite facies after coesite transfers into quartz) and *T*=693–761 °C based on the geothermometer of Ravna^40^. *In situ* phengite dehydration melting resulted in MS inclusions of Kfs+barium-bearing Kfs+Pl in garnet, connected by 4–10 μm wide veinlets consisting of Bt+Kfs+Pl next to the phengite (Fig. 4e). MS inclusions of Kfs+Qz (Fig. 4f) and cross-cutting Kfs veins are also present in this stage.

#### Eclogitic residue

In this terminal stage, most of the melted eclogite shows evidence of strong amphibolite-facies retrogression. The best preserved mineral assemblage of eclogitic residue is Grt+Omp+Qz+Rt, with large amounts of symplectite replacing omphacite, and phengite completely disappears from this phase. The P–T estimation for this stage is *P*=0.8–1.4 GPa, *T* (average)=669–703 °C based on the geothermometer of Ravna^40^. MS inclusions of Kfs+Qz sit in garnet from eclogitic residue in General's Hill (Fig. 4g). Interstitial cuspate veinlets of plagioclase+K-feldspar with very low dihedral angles first form isolated ‘strings of beads’ of melt along grain boundaries and triple junctions of quartz (Fig. 4h, i), and with higher degrees of melting, eventually forming interconnected 3D networks along grain boundaries in the leucosome at General's Hill (Fig. 4j–l), allowing the melt to escape from the intergranular realm and collect in low-stress areas as documented at the macroscopic scale in stages I–II above.

MS inclusions within omphacite and garnet from UHP eclogites have been recently reported as limited evidence for local and minor phengite dehydration-related melting of UHP eclogite^22^,^43^,^44^,^45^. We provide further and stronger evidence from the closely related outcrops at Yangkou and General's Hill (Fig. 1). The MS inclusions at these two locations range from K-feldspar+quartz to intermediate types consisting of K-feldspar (including barium-bearing K-feldspar)+quartz±silicate (plagioclase, epidote or diopside)±barite within omphacite and garnet from four metamorphic stages of eclogite. Our new microstructural evidence from Yangkou and General's Hill demonstrates more advanced melting of eclogite.

First, *in situ* phengite dehydration melting formed MS inclusions in UHP eclogite and HP quartz eclogite (Fig. 4d–f), suggesting *in situ* melting and coexistence of aqueous fluids with hydrous melts under HP eclogite facies condition^44^ when retrogression of phengite and omphacite were promoted by partial melting. Second, MS inclusions in garnet from eclogitic residue and interstitial cuspate veinlets of plagioclase+K-feldspar+epidote merged to form interconnected 3D networks along grain boundaries in the residue and leucosome at General's Hill (Fig. 4g–l), allowing the melt to escape from the intergranular realm and collect in low-stress areas as documented at the macroscopic scale in stages I–II and shown in Fig. 1. Third, intergranular multi-solid phase K-feldspar+quartz in UHP stage 1 and 2 eclogite are surrounded by radial fractures and cut through by K-feldspar veins (Fig. 4b,c). This indicates that later stage of veins produced by melting cross-cutting the UHP eclogites, with limited fluid/melt accompanying this event, replacing the earlier intergranular coesite with Kfs+Qz. All of these features are diagnostic of partial melting^6^,^38^,^46^, indicating clearly that the UHP eclogite at Yangkou and residue in General's Hill melted in progressive multiple stages during their retrograde ascent from UHP conditions.

**Figure 7:**
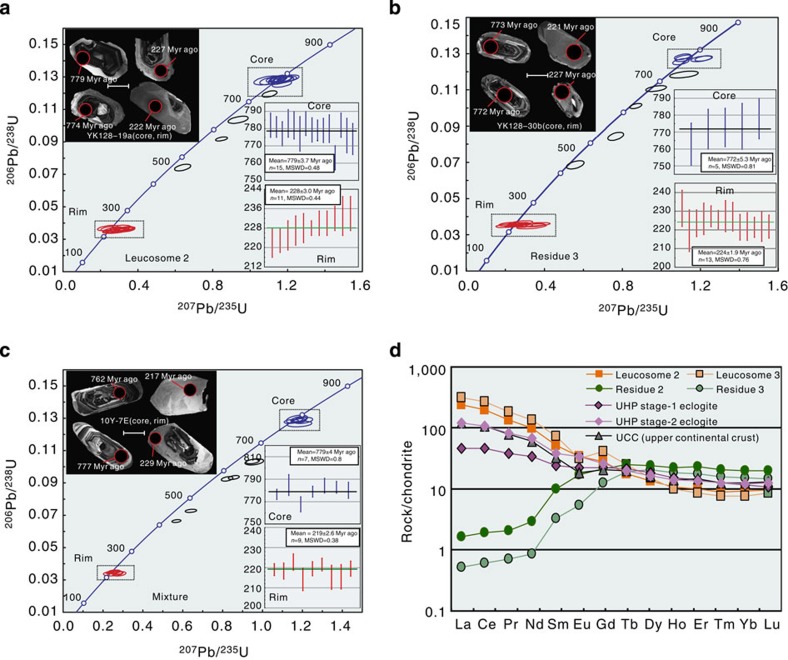
Concordia diagrams and REE plots. (**a**) Concordia diagram and weighted average age of one leucosome sample with representative zircon CL images. (**b**) Concordia diagram and weighted average age of one residue sample with representative zircon CL image. (**c**) Concordia diagram and weighted average age of one mixture sample contains both leucocratic and residual material (melting process stage II) with representative zircon CL image. (**d**) Chondrite-normalized REE patterns of two representative leucosome samples, two residue samples, one UHP stage-1 eclogite and one UHP stage-2 stage eclogite. Compositionally, the REE of the leucosome are comparable with the standard upper continental crust (UCC) by Rudnick & Gao^48^. See Supplementary Data Set 4 and 5 for original data and analyses. The weighted average age calculation is based on the ^206^Pb/^238^U since the formation age is younger than 1,000 Myr ago. Age data processing was carried out with ISOPLOT software, the error ellipses are the absolute error value and 1*σ*. Scale bars in the zircon CL images are 50 μm across.

Experimental work on the partial melting of phengite-bearing eclogite indicates that the P–T condition of melting was ~1.5–2.0 GPa, T≤800–850 °C (ref. 24). However, *in situ* partial melting of phengite in natural samples is observed at the quartz eclogite metamorphic stage when all the coesite completely transformed into quartz, which is between the UHP stage and amphibolite-facies stage. Therefore, melting started from quartz-eclogite stage (lower limit, *P*=1.5–2.8 GPa, *T*=760 °C, about 90–50 km depth) and continued through lower-grade conditions (amphibolite facies, about 30 km depth) for about 9 Myr (Fig. 6), involving higher degrees of melting, and eventual interaction with melts derived from the country rocks. On the basis of the geochronological estimation of UHP and amphibolite facies metamorphic stages^12^, this timing is between 230 and 215 Myr ago, corresponding to the age of 228–219 Myr ago we report from zircon dating of leucosome, and residue samples (Fig. 7a–c) from General's Hill.

Thus, evidence from microscopic to outcrop scales at Yangkou and General's Hill clearly shows that partial melting of the eclogite began at the intergranular scale as imaged in SEM, the melts merged along a 3D grain boundary network then flowed along micro cracks (Fig. 4e–i), then into macroscopic structures such as extensional shear bands and pressure shadow regions in folds. These melt pockets were then able to coalesce into melt channels and dikes, releasing the melt from the system during widespread partial melting of the eclogite.

### Geochronology and geochemistry of melting eclogite

Zircons from leucosome (YK128-19a, same as leucosome 2 in Fig. 7d), residue (YK128-20b, same as residue 3 in Fig. 7d) and mixture (10Y-7E, stage II) show euhedral and prismatic shapes, exhibit distinct core-rim structures in cathodoluminescence (CL) images (Fig. 7). Cores are characterized by oscillatory zoning and rims show grey unzoned luminescence in CL images (Fig. 7). U–Pb ages of zircons analyzed by laser ablation-inductively coupled plasma mass spectrometer (LA-ICP-MS) are summarized in Supplementary Data Set 4 and shown in Fig. 7a–c. Most of the inherited cores record concordant U–Pb ages that yield a weighted average ^206^Pb/^238^U age of 770–780 Myr ago (Fig. 7), and zircon rims from the leucosome show concordant U–Pb ages and yield a concordia age of 228±3.0 Myr ago (1*σ*, *n*=11) (Fig. 7a), although a few scattered data have ages of ca. 400, 500 and 700 Myr ago. The latter few ages might be related to Pb loss due to incomplete resetting before zircon experienced peak metamorphism^3^. Rims from the mixture sample yield an age of 219.3±2.6 Myr ago (1*σ*, *n*=9) (Fig. 7b), and zircon rims from the residue yield an age of 224±1.9 Myr ago (1*σ*, *n*=13) (Fig. 7c). We interpret these ages to mean that the protolith has an age of 780 Myr and the partial melting stages lasted about 9 Myr ago from 228 to 219 Myr ago, shortly after UHP metamorphism at circa 230 Myr ago^12^,^13^.

A wide spectrum of REE patterns and variations in major and trace element abundances in both the leucosome and residue suggest a continuous melting event (Fig. 7d; Supplementary Data Set 1 and 5; Supplementary Fig. 2) that lasted about 9 Myr, consistent with the field and microstructural data presented above. We interpret the peak eclogite sample as the closest representative of the mafic protolith that was subducted, metamorphosed and partially melted. This eclogite is characterized by a LREE-enriched pattern (La/Sm_cn_=1.97; Gd/Yb_cn_=1.94; La/Yb_cn_=4.09) and negative Nb (Nb/Nb*=0.39) but positive Pb (Pb/Pb*=2.71) anomalies (Supplementary Fig. 2). It is difficult to distinguish between eclogite melting in subducted mid-ocean ridge basalt and eclogite melting in the SCLM. However, depletion of Nb (negative Nb anomaly) is more consistent with a SCLM source than subducted mid-ocean ridge basalt. Normalized Gd/Yb values imply that garnet residue in the source with partial melting likely have taken place above the garnet stability field^47^. Alternatively, superchondritic Gd/Yb_cn_ ratios might also have been inherited from the SCLM source.

Two enriched end-members of leucosome (leucosomes 2 and 3) and two-depleted end-members of residue (residues 2 and 3) have complementary REE patterns (Fig. 7d). The leucosome end-members have the following trace element characteristics: (1) strongly enriched LREE patterns (La/Yb_cn_=26.4–42.7); (2) large negative Nb (Nb/Nb* =0.07–0.08) and Ti (Ti/Ti*=0.05–0.14) anomalies; and (3) positive Pb anomalies (Pb/Pb*=2.9–6.2) (Fig. 7d and Supplementary Data Set 1 and 5). In addition, the leucosome is strongly enriched in SiO_2_ (70.9–76.8 weight percent (wt.%)) and large ion lithophile elements (LILE) but depleted in MgO (0.98–1.74 wt.%) and transition metals (Cr, Ni and Co), in comparison with the residue and peak eclogite (MgO=5.32 wt.%) (Supplementary Data Set 1). Compositionally, the leucosome is comparable to the upper continental crust^48^, suggesting a genetic link between subduction zone geodynamic and petrogenetic processes and the origin of continental crust.

The residual end-members display strongly depleted LREE (La/Sm_cn_=0.15–0.16; La/Yb_cn_=0.03–0.04) patterns and positive Nb (Nb/Nb*=12.2–58.3) and Ti (Ti/Ti*=1.20–2.36) anomalies, consistent with extraction of LREE by melts and retention of Nb and Ti by rutile in the residue (Fig. 7d and Supplementary Data Set 1). In addition, the residue gained MgO (6.3–6.8 wt.%), transition metals and heavy REE relative to peak eclogite. Collectively, REE systematics of leucosome and residue are consistent with extraction of leucosome melts in the garnet and/or hornblende stability field^47^.

## Discussion

Documentation of partial melting of eclogite has important implications for global tectonic processes. Here, we outline a few of the salient implications for lithospheric tectonic processes.

The presence of rheologically weak melt channels in eclogite subducted to >200 km depth significantly changes current ideas about the strength of eclogitic slabs in deep continental or oceanic subduction zones^49^. These melt channels allow flow and movement between different 10 to 20 m scale lenses of eclogite, and when integrated over the thickness of the entire subducting slab allow significantly different behaviour than expected for unmelted eclogite. For instance, if eclogite in the deep lithosphere is partially melted with melt channels, and in the lower crust or upper mantle, then lateral flow of deep crust/upper mantle might be accommodated along melt channels. Lower-crustal flow channels have been postulated to exist in several places including eastern Asia, allowing deep crust to laterally escape from the India-Asia collision zone^26^,^50^. We suggest that the mechanism that allows this flow is the formation of melt channels similar to those documented here, significantly changing the rheology of the lower crust and upper mantle.

**Figure 8:**
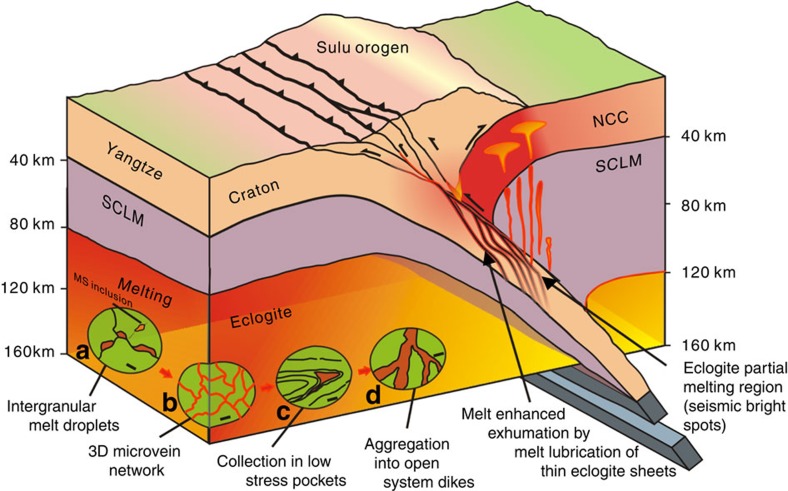
Model showing partial melting of subducted eclogite and how melt channels aid exhumation, feed crustal lavas and make seismic bright spots. The elliptical insets **a**–**d** represent the progressive different stages and scales of partial melting of eclogite and melt segregation during exhumation. Eclogite begins partial melting by initial melt droplets forming along grain boundaries (**a**, scale bar, 50 μm), which then coalesce into 4–10 m wide intergranular veinlets (**b**, scale bar, 1 cm), which then move along foliation planes and extensional shear zones, eventually forming larger melt pockets in low-stress zones such as fold hinges between units with different rheologies (**c**, scale bar, 10 cm). Melts in these pockets then merge through interaction of melting and deformation, enhancing deformation in these zones, forming melt channels consisting of ~50% melt and 50% residual eclogite (**d**, scale bar, 1 m). Where these melt channels merge melts escape and form metre-scale dikes that may interact with melts derived from the gneisses and transport magma to higher lithospheric levels. SCLM, subcontinental lithospheric mantle; NCC, North China Craton.

Further, the presence of thin melt channels in deeply subducted lithosphere can significantly change the conditions for exhumation of UHP metamorphic rocks. There has been a long-standing debate about how thin slices of UHP rocks can be exhumed from 200 km to the surface without being retrogressed to lower grade assemblages. The melt channels in eclogite may have lubricated the edges of exhumed slices of UHP rocks (Fig. 8), allowing them to be rapidly transported to the surface without time to transform into lower grade mineral assemblages^33^.

Peak UHP metamorphism in Sulu eclogites (recording depths of possibility up to 200 km) occurred about 230 Myr ago^12^,^13^, and the partial melts we report here have ages of 228–219 Myr ago, showing that the melting occurred shortly after the peak UHP event, while the eclogites were still at great depth and began their rapid exhumation to the surface (Figs 6 and 8). A persistent controversy in geosciences is whether or not subducted slabs can partially melt, and generate magmas such as adakites that contribute to arc magmatism^51^,^52^. Our study at General's Hill represents the first documented case in the world where deeply subducted eclogites can be conclusively shown with field, microstructural and geochemical evidence to have undergone partial melting, generating felsic magmas that migrated upwards, potentially contributing to arc magmatism and the formation of continental crust at convergent plate boundaries. The felsic melts have a dacitic composition (see Fig. 5), consistent with melting from both a subducted slab and the SCLM. Interestingly, Late Triassic plutons with ages similar to the age of melting eclogite in this study crop out 150 km to the northeast of the study area, and have geochemical affinities consistent with derivation by melting of mafic and felsic rocks of the deeply subducted north margin of the Yangtze craton^53^. We suggest that the source of these and other related plutons has now been identified (Fig. 8).

One of the enigmatic features of some deep-crustal seismic reflection profiles is the presence of ‘bright zones’ interpreted to represent areas of melt, high fluid content or unusual rock compositions^25^,^26^. The deeply subducted, partially melted eclogites from General's Hill show that eclogites can develop regularly spaced melt channels, a metre or two thick, that would act as significant seismic anomalies (Figs 1c, 2, and 8). Field observations from General's Hill thus resolve a long-lived controversy in seismology and provide a link between field geology, structural analysis, geophysics, geochronology and crustal evolution.

## Methods

### Mineral major element analyses

Mineral major element analyses were performed on JEOL JXA-8800 and JEOL JXA-8100 electronic microprobes (EMPs) at the Institute of Mineral Resources, Chinese Academy of Geological Sciences and the Key Laboratory of Submarine Geosciences, State Oceanic Administration, respectively. The working conditions of both were as follows: 15 kV acceleration voltage with 20 nA beam current and 5 μm beam spot.

### Whole-rock major and trace element analyses

Whole-rock samples were crushed in a corundum jaw crusher (to 60 meshes). About 60 g of each sample was powered in an agate ring mill to >200 meshes for major and trace elements. Two samples, including UHP stage-1 eclogite (12YK5-2) and stage-2 eclogite (12YK8-1) were analyzed in the Comprehensive Rock and Mineral Test Center, Wuhan, China. Other rock samples were analyzed at the State Key Laboratory of Geological Processes and Mineral Resources (GPMR), China University of Geosciences (Wuhan). Whole-rock major element compositions were measured by a Shimadzu XRF-1800 sequential X-ray fluorescence spectrometer, the detailed experimental processes and conditions are described by Ma *et al*.^54^ The major elements were analyzed by a wet chemical method according to the GB/T 14506.28-1993 standard, whereas the analytical standards of the H_2_O+, CO_2_ and LOI were measured according to the GB/T14506.2-1993 standard, the GB 9835-1998 standard and the LY/T 1253-1999 standard, respectively.

Whole-rock trace elements were analyzed by an Agilent 7500a ICP-MS, the detailed sample-digestion procedure for ICP-MS analyses, analytical precision and accuracy for trace elements including REE, HFSE, LILE and transition metals following the protocols of Liu *et al*.^55^ About 50 mg samples were digested by HF+HNO_3_ in Teflon bombs for ICP-MS analysis. Sample dissolution was conducted under super clean lab conditions. International standards AGV-2, BHVO-2, BCR-2, RGM-1 and GSR-1 were used as reference materials to estimate analytical precision. All the REE plot figures are chondrite normalized, the normalized value is based on Sun and McDonough^56^.

### Mineral identification and zircon CL imaging

Back-scattered electron imaging, X-ray energy dispersive spectroscopy for mineral identification and zircon CL imaging were acquired on a FEI Quanta 450 field emission gun SEM with an attachment of Oxford SDD Inca X-Max 50 energy dispersive spectroscopy and Gatan Mono CL4^+^ CL system. The working conditions for SEM imaging were 20 kV with a spot size of ~6.0 μm and working distance of ~12 mm. For zircon CL imaging, the working conditions were set to be 10 kV with a spot size of ~5 μm and working distance of ~14 mm.

### Raman spectra analyses

Raman spectra on the intergranular coesite were obtained by a Renishaw RM 1000 Raman Spectrometer with 3.4 mV of 514 nm Ar laser excitation at room temperature. The beam size for Raman spectroscopy was about 1.5 μm.

### LA-ICP-MS U–Pb dating

LA-ICP-MS U–Pb dating, zircon U–Pb dating and trace element analyses were conducted synchronously by LA-ICP-MS, and detailed operating conditions for the laser ablation system and the ICP-MS instrument, analytical procedures and data processing are the same as described by Liu *et al*.^55^,^57^ Laser sampling was conducted using a GeoLas 2005 System with a spot size of 32 μm. Laser repetition rate was set at 6 Hz with energy density of 60 mJ. Each analysis includes ~20–30 s background acquisition, followed by 30–50 s data acquisition from zircon samples. The zircon standard 91500 was used as an external standard to calibrate isotope fractionation, which was analyzed twice for every five analyses. Zircon standard GJ-1 was analyzed as an unknown. NIST610 was also applied to correct the time-dependent drift of sensitivity and mass discrimination for the trace element analysis. Off-line selection, integration of background and analytical signals, time-drift correction and quantitative calibration were conducted by the software of ICPMSDataCal^55^.

## Author contributions

L.W. and T.M.K. designed the project, mapping, collected and interpreted data and wrote the paper. L.W. designed the lab work and finished SEM, electronic microprobe, CL and part of ICP-MS work with student S.J.W. and J.Fu. A.P., K.Q.Z. and J.P.W. participated in part of the field work and collected some of the samples for geochemistry and age analysis, and A.P. guided the dating and trace element analysis. X.F.J. and H.D. assisted with the geochronology and formatting and drafting of Figure 6 and the Supplementary Information.

## Additional information

**How to cite this article**: Wang, L. *et al*. Partial melting of deeply subducted eclogite from the Sulu orogen in China. *Nat. Commun.* 5:5604 doi: 10.1038/ncomms6604 (2014).

## Supplementary Material

Supplementary Figures Supplementary Figures 1-2

Supplementary Dataset 1 Major and trace element composition of rock pairs (leucosome, residues), UHP

Supplementary Dataset 2 Yangkou Bay/Diaolongzui leucosomes are quartz (29-51 wt. %), anorthite (3-12

Supplementary Dataset 3 EPM data for P-T estimations of UHP eclogite and

Supplementary Dataset 4 LA-ICP-MS zircon U-Pb data of leucosome, residue and partially melted eclogite

Supplementary Dataset 5 Gain and loss calculations indicate that both the residues and leucosomes are enriched
