# Supplementary material for: Partial melting of deeply subducted eclogite from the Sulu orogen in China
Source: Nat Commun. 2014 Dec 17;5:5604. doi: 10.1038/ncomms6604 (PMC4284643; doi:10.1038/ncomms6604)
Supplement: Supplementary Figures — 1-2 [file ncomms6604-s1.pdf]

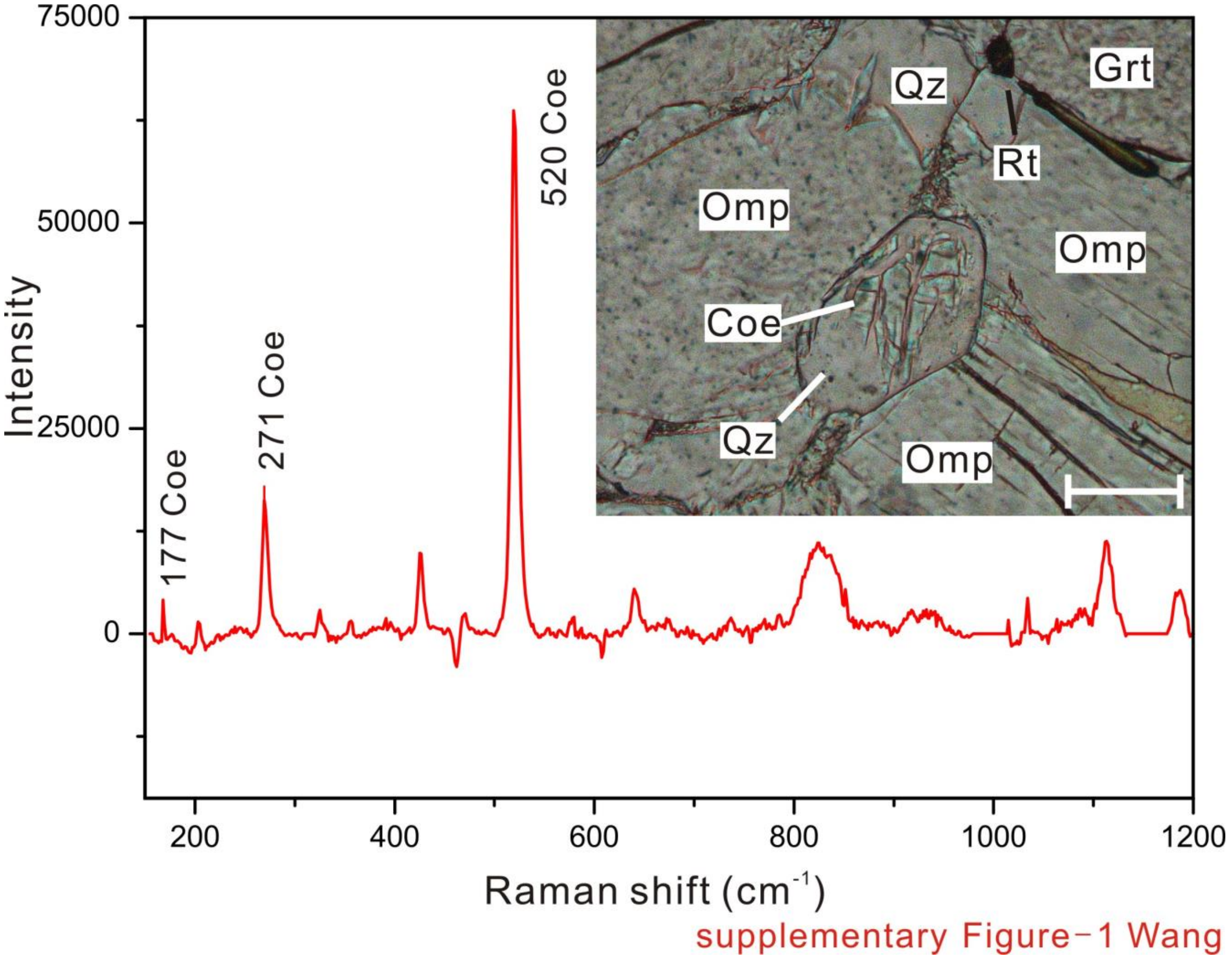

Supplementary Figure 1. Raman spectrograph and microphotograph of intergranular coesite within UHP eclogite. Sample 12YK5-2a, UHP-stage 1 eclogite. Scale bar in the microphotograph, 50μm.

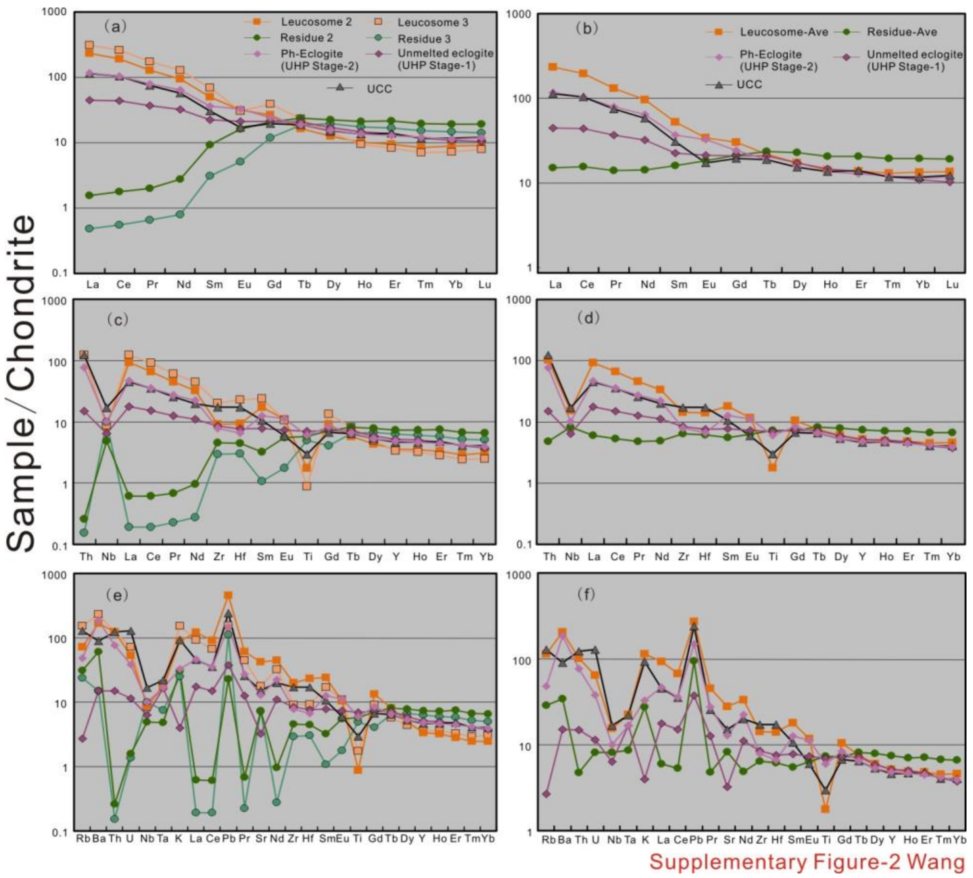

Supplementary Figure 2. Plots of data from Supplementary Dataset 5. This figure shows a wide variety of REE and trace element abundances in both the leucosome and residue, suggesting multiple melt extraction events over a prolonged period (about 9 Ma according to the geochronology).
